# “Restless Nights, Stressed Hearts”: The Link Between Sleep Disorders and Takotsubo Syndrome—A Comprehensive Review

**DOI:** 10.31083/RCM28244

**Published:** 2025-05-06

**Authors:** Ioannis Alevroudis, Magda Petridou, Agni Sakkou, Serafeim-Chrysovalantis Kotoulas, Sotirios Matzolas, Panagiotis Roumelis, Maria Stougianni, Eleni Massa, Eleni Mouloudi

**Affiliations:** ^1^Adult ICU, General Hospital of Thessaloniki “Ippokrateio”, 54642 Thessaloniki, Greece; ^2^Third Department of Cardiology, General Hospital of Thessaloniki “Ippokrateio”, Aristotle University of Thessaloniki, 54642 Thessaloniki, Greece

**Keywords:** insomnia, obstructive sleep apnea, restless leg syndrome, sleep deprivation, Takotsubo syndrome, hypothalamic-pituitary-adrenal (HPA) axis, elevated catecholamine levels, heart rate variability

## Abstract

Takotsubo syndrome (TTS), also known as stress-induced cardiomyopathy or “broken heart syndrome”, is characterized by transient left ventricular dysfunction, often triggered by emotional or physical stress. Emerging evidence suggests that sleep-disordered breathing (SDB) and sleep disruption may play a significant role in the pathophysiology and exacerbation of TTS. This review explores the influence of conditions such as obstructive sleep apnea (OSA), insomnia, and other sleep disturbances on the onset and progression of TTS. SDB, particularly OSA, is marked by repetitive episodes of upper airway obstruction during sleep, leading to intermittent hypoxia and increased sympathetic nervous system activity. These physiological changes can trigger or exacerbate TTS by promoting myocardial stress and impairing autonomic regulation. Insomnia and other forms of sleep disruption also contribute to heightened sympathetic activity and elevated stress hormone levels, which may precipitate TTS in susceptible individuals. Thus, this review synthesizes current research on the mechanisms linking sleep disturbances to TTS, highlighting the impact of nocturnal hypoxia, sleep fragmentation, and autonomic dysregulation. Moreover, this review discusses the clinical implications of these findings, emphasizing the need to screen and manage sleep disorders in patients with or at risk of TTS. Addressing sleep disturbances through therapeutic interventions may reduce the incidence and recurrence of TTS, offering a novel approach to managing this condition. In conclusion, this review underscores the importance of recognizing and treating SDB and sleep disruption as potential contributors to Takotsubo syndrome. Future research should focus on elucidating the precise mechanisms involved and determining effective strategies for integrating sleep management into the care of patients with TTS.

## 1. Introduction

Takotsubo syndrome (TTS), often referred to as “broken heart syndrome”, is a transient form of cardiomyopathy typically triggered by acute emotional or physical stress. First described in Japan in the early 1990s, TTS derives its name from the Japanese word for an octopus pot (“takotsubo”), as the left ventricle of the heart assumes a similar shape during the acute phase of the syndrome [1]. While traditionally associated with emotional stressors, recent research has drawn connections between sleep disturbances and the development of TTS. Given the increasing prevalence of sleep disorders such as insomnia, obstructive sleep apnea (OSA), and restless leg syndrome (RLS) in modern societies, understanding their potential influence on TTS is crucial.

This review examines the current body of literature exploring the relationship between sleep disorders and TTS, highlighting the pathophysiological mechanisms, clinical outcomes, and potential therapeutic strategies.

## 2. Epidemiology of TTS

The incidence of TTS in USA and Europe is calculated 50,000 to 100,000 per annum [2]. Having similar symptoms with acute coronary syndrome (ACS), it represents about 1–2% of the final diagnosis after a thorough investigation has taken place [3]. The syndrome is highly prevalent among women, with 89.8% of all cases occurring in females, who have a mean age of 66.8 years [4]. In the male population TTS incidence is much lower at the expense of the mortality which is higher than the female gender [5].

## 3. Pathophysiology of TTS 

TTS is characterized by a temporary weakening of the heart’s left ventricle, primarily driven by excessive activation of the predominantly sympathetic nervous system (SNS). Elevated catecholamine levels are a key trigger, leading to myocardial stress and dysfunction [6]. Several stressors were found to precede the onset of TTS in a considerable number of patients [7]. Emotional or psychological stress triggered by events such as the sudden loss of a loved one or friend, fear, natural disasters, or intense physical exertion is often observed prior to its onset [8]. Newer data however, show that TTS could be the result of a positive life event, altering the name of the syndrome into a happy heart syndrome [9]. Finally, there is a 20 percent of patients who do not report any exposure to stress prior to the TTS development. While most patients recover within weeks, the syndrome can be life-threatening, especially in the acute phase, when complications such as heart failure, arrhythmias, or even cardiogenic shock can occur.

Elevated norepinephrine levels have been found in the coronary sinus of patients with TTS, indicating an increased release of myocardial catecholamines [10]. Consequently, the different ballooning patterns and clinical symptoms of TTS can be triggered by administering beta-agonists and catecholamines that led immediately to the development of TTS [11]. However, adrenaline might instead serve as a trigger for localized disruption of cardiac sympathetic function [12, 13].

Overall, heightened sympathetic stimulation is vital to TTS. Currently, the hypothesis of the involvement of a catecholamine surge is the most accepted. However, the precise mechanism through which a surge in catecholamines leads to myocardial stunning and the varied patterns of regional ballooning is still unclear.

Multivessel coronary spasm resulting from sympathetic hyperactivity has been suggested as a potential underlying mechanism in TTS [14]. In fact, mental stress has been shown to trigger endothelial dysfunction, which can be mitigated through the use of endothelin-A receptor antagonists [15].

Patients with TTS show a greater prevalence of coronary artery tortuosity, a longer recurrent wraparound left anterior descending artery (LAD), and recurrent LAD segments compared to matched controls [16, 17]. Plaque rupture, thrombosis, and subsequent transient ischemia followed by rapid lysis have been suggested as potential mechanisms for myocardial stunning in TTS. Medical literature reports that TTS patients have epinephrine, norepinephrine, and dopamine levels that are 7 to 34 times higher than established normal ranges [18].

The sudden release of catecholamines from sympathetic nerves, the adrenal medulla, or as a result of drug therapy can cause temporary but prolonged left ventricular dysfunction, often accompanied by secondary myocardial inflammation. This happens because coronary microcirculation and cardiac myocytes are highly sensitive to stress hormones [19]. TTS is a condition in which the release of stress hormones, mediated by the central nervous system, is triggered by acute brain injury. This injury can be physical, such as intracranial bleeding or head trauma, or psychological, such as sudden emotional stress [20].

Most TTS patients exhibit angiographically normal coronary arteries or non-obstructive coronary artery disease (CAD). As a result, coronary microcirculation is believed to play a central role in the development of TTS.

The coronary microcirculation, comprising pre-arterioles and arterioles with diameters under 500 µm, controls blood flow by responding to mechanical, metabolic, and neural factors. Dysfunction in this system may lead to reduced myocardial perfusion [21]. For instance, the vasoconstrictive effects of catecholamines are mainly observed in the coronary microvasculature, where α1-receptors are predominantly expressed [22]. Interestingly, a more recent analysis found that, despite worse left ventricular (LV) systolic function, acute coronary flow reserve (CFR) was less compromised in TTS compared to acute myocardial infarction (AMI). This suggests that mechanisms other than reduced CFR contribute to the development of regional wall motion abnormalities (RWMA) in TTS [23].

Chronic psychosocial or traumatic stress can lead to persistent hyperactivity of the hypothalamic-pituitary-adrenal axis (HPAA), ultimately resulting in chronic hypoactivity [24, 25]. Additionally, the direct impact of catecholamines on cardiomyocytes can cause transient dysfunction of the left ventricle [26]. Patients who have experienced TTS exhibit increased vascular reactivity and impaired endothelial function in response to acute mental stress [27]. In summary, elevated microvascular reactivity, likely driven by the sympathetic nervous system, should be considered a central factor in the development of TTS.

Earlier studies have uncovered underlying psychiatric conditions, including depression, anxiety, mania, and psychosis, as well as neurological disorders such as subarachnoid hemorrhage, stroke, transient ischemic attack, seizures, and pheochromocytoma, all of which can trigger stress and physiological alterations. The cortisol-induced release of catecholamines may also contribute to the pathogenesis of TTS [28].

General anxiety levels are significantly higher in healthy individuals compared to TTS patients, whereas illness-related anxiety is particularly common among those with TTS [28]. Patients with depression and anxiety show increased levels of microRNA 16 and 26a. In a rodent model where these microRNAs were overexpressed, the administration of exogenous epinephrine was associated with apical wall motion abnormalities [29, 30].

Some researchers suggest that adrenoreceptor stimulation can disrupt the equilibrium between oxygen supply and demand, causing myocellular hypoxia. This hypoxia is worsened by metabolic changes and electrolyte imbalances resulting from altered membrane permeability, potentially contributing to myocardial toxicity. Thus, TTS may affect the autonomic nervous system in a more intricate way than just a sudden increase in catecholamines.

For many patients, psychological stress serves as a key trigger for TTS, even in the context of a physical illness that may also induce psychological stress [31].

Given the high prevalence of TTS in postmenopausal women, a hormonal influence can be suggested. Research indicates that decreased estrogen levels following menopause may increase susceptibility to TTS in women [32]. Estrogens have been demonstrated to attenuate the sympathetic response to mental stress in perimenopausal women and to decrease vasoconstriction induced by catecholamines [33, 34].

**Fig. 1.**
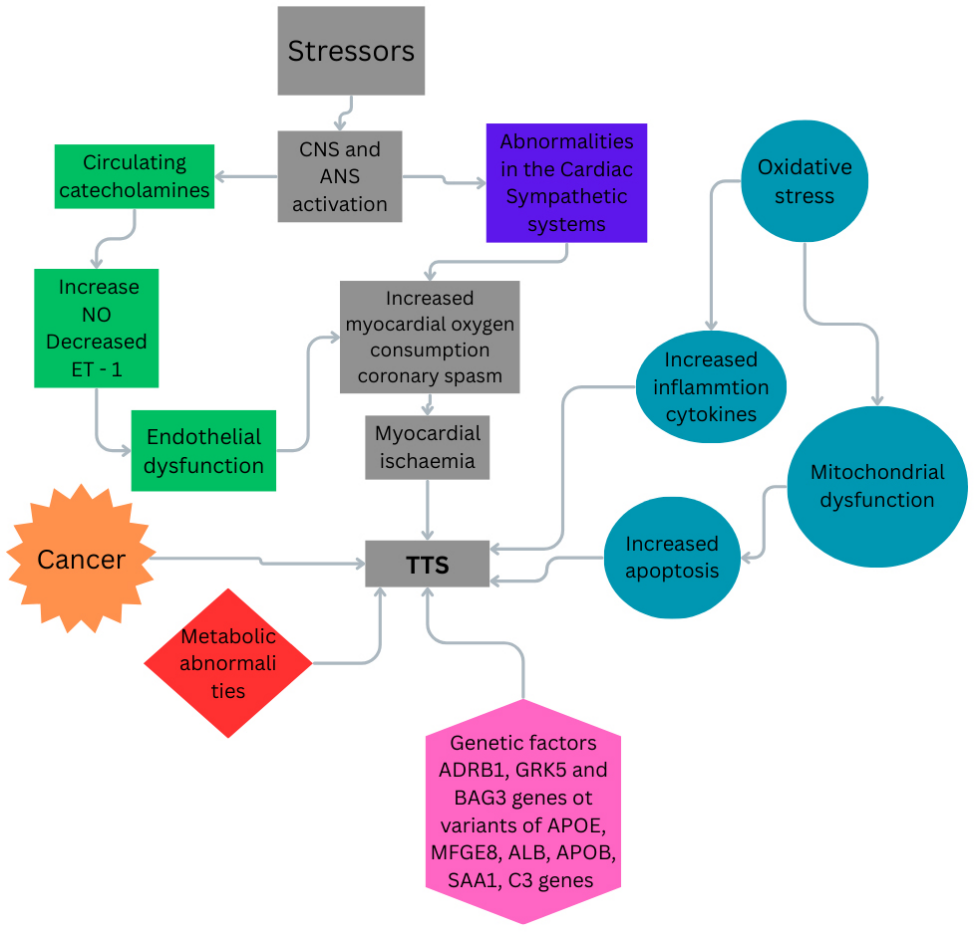
**The possible mechanisms related to TTS**. TTS, takotsubo syndrome; CNS, central nervous system; ANS, autonomic nervous system; NO, nitric oxide; ET-1, endothelin-1. Fig. 1 was created in the canva.com program.

TTS is marked by the infiltration of macrophages into the myocardium, shifts in the distribution of monocyte subsets, and heightened levels of pro-inflammatory cytokines in the plasma. Notably, elevated serum levels of pro-inflammatory cytokines, including C-X-C motif chemokine ligand 1 (CXCL1), interleukin (IL)-6, and IL-8, have been reported. The authors observed a rise in pro-inflammatory cell surface antigen CD14++CD16– macrophages, coupled with a decline in intermediate CD14++CD16+ and non-classical CD14+CD16++ monocytes. While ultrasmall superparamagnetic iron oxide (USPIO) enhancement disappeared after five months, persistently elevated IL-6 levels and a reduced count of intermediate monocytes suggested a chronic low-grade inflammatory state [35]. In Fig. 1, is presented the wide variety of mechanisms that lead to the TTS.

In summary, inflammation is a key factor in the pathophysiology of TTS. Understanding the underlying pathogenesis and pathophysiology is essential for developing effective treatments for acute episodes and preventing long-term recurrent events.

## 4. Sleep Disorders: An Overview

Sleep disorders include a wide range of conditions that interfere with normal sleep patterns.

The most common sleep disorders include:

(a) Insomnia: Characterized by difficulties in initiating or maintaining sleep.

(b) OSA: A condition characterized by repeated interruptions in breathing during sleep caused by airway obstruction.

(c) RLS: A neurological condition that triggers an irresistible need to move the legs, frequently disturbing sleep.

(d) Sleep deprivation.

An increasing amount of evidence suggests that sleep disturbances, through similar mechanisms that contribute to cardiovascular risk, may play a significant role in the onset of TTS. Research has identified multiple ways in which sleep disorders, particularly OSA and insomnia, might predispose individuals to TTS.

## 5. Sleep Disorders as a Cardiovascular Risk Factor

### 5.1 Insomnia and TTS

As defined by the DSM-V1 (diagnostic and statistical manual of mental disorders) [36], insomnia involves dissatisfaction with sleep quantity or quality, accompanied by one or more of the following symptoms: trouble falling asleep, difficulty staying asleep (frequent awakenings), or waking up too early. These symptoms must occur at least three times per week for a period of three months, even when adequate opportunities for sleep are available.

Insomnia can be classified into acute (short-term) and chronic (lasting longer than three months) and is influenced by genetic, environmental, and psychological factors. Women and persons of older age are more frequently afflicted [37]. Chronic insomnia often arises from heightened emotional arousal, a disrupted circadian rhythm, or an overactive hypothalamic-pituitary-adrenal (HPA) axis. Vgontzas and Chrousos highlight that chronic insomnia affects the HPA axis, resulting in elevated cortisol levels that correlate with cardiovascular health risks, and insomnia’s impact on cardiovascular health could predispose individuals to TTS [38]. This overactivity of the HPA axis results in elevated nighttime cortisol and adrenaline levels, reduced nocturnal melatonin secretion, leading to prolonged wakefulness and difficulty initiating sleep. These disruptions impair the circadian rhythm and contribute to fragmented sleep, further exacerbating the condition. Chronic insomnia is also associated with increased sympathetic nervous system activation, leading to higher resting heart rates, blood pressure elevations, and elevated catecholamine (stress hormone) levels, which collectively can strain cardiovascular health [39, 40, 41]. Research on this link suggests that the chronic elevation in stress hormones and increased sympathetic activity in insomnia can predispose individuals to acute cardiovascular events under stress [42]. In a large population-based study, Laugsand *et al*. [43] found that individuals with chronic insomnia had a significantly higher risk of developing cardiovascular diseases, including cardiomyopathy.

The connection between insomnia and TTS is rooted in their shared stress-related origins. Insomnia is often accompanied by autonomic dysregulation, marked by increased sympathetic activity and reduced parasympathetic activity, which creates a state of chronic physiological stress. This prolonged activation of the sympathetic nervous system may contribute to higher circulating levels of catecholamines, a primary factor implicated in the onset of TTS [44]. A study by Jarrin *et al*. [45] explored insomnia’s impact on cardiovascular autonomic regulation and concluded that poor sleep quality is associated with increased sympathetic tone and diminished heart rate variability, indicators of cardiovascular strain that mirror the autonomic dysfunction seen in TTS.

Hyperarousal is a key pathophysiological hypothesis in insomnia research. It suggests that a state of cognitive, emotional, and physiological hyperarousal persists both during the day and night, serving as a significant cause and sustaining factor for the condition [46]. The chronic physiological arousal associated with insomnia is thought to exacerbate mood disorders by disrupting neurochemical signaling and increasing cortisol levels. This bidirectional relationship between poor sleep and psychiatric symptoms further complicates both insomnia and its comorbidities, creating a cycle of sleep disruption and mental distress [47, 48, 49, 50, 51, 52]. Insomnia is a significant predictor of future mental illness. It can also arise as a result of or coexist with other psychiatric or medical conditions, especially depression and anxiety disorders. This cyclical relationship may further elevate the risk of developing conditions like depression, which is itself a recognized risk factor for cardiovascular disease and TTS. Additionally, depression can worsen the physical strain on the heart, creating a harmful feedback loop [53]. Therefore, insomnia should be considered a chronic condition with widespread effects on multiple physiological systems.

Insomnia raises the risk of hypertension (HTN), heart failure (HF), and coronary heart disease, particularly when sleep duration is less than 6 hours [54]. Additionally, the more insomnia symptoms a person experiences, the higher their risk of developing heart failure [55].

Future research should focus on identifying specific markers in individuals with insomnia that may predispose them to TTS and investigating whether treating insomnia can reduce the incidence of stress-induced cardiomyopathy. Longitudinal studies could further elucidate whether insomnia independently increases the risk of TTS or if its impact is primarily mediated through stress sensitivity and autonomic dysregulation.

While specific studies linking insomnia and TTS are limited, the pathophysiological similarities between the two conditions make a compelling case for further research in this area.

### 5.2 Obstructive Sleep Apnea and TTS

The relationship between sleep disturbances and cardiovascular health is well-documented.

There are three main apnoea types:

(1) OSA

(2) Central sleep apnoea (CSA)

(3) Complex sleep apnoea (a combination of OSA and CSA)

An apnea is characterized by a complete cessation of breathing lasting at least 10 seconds or longer, while a hypopnea refers to a partial reduction in breathing for the same duration. The mechanism behind obstructive apnea involves the relaxation and backward displacement of the genioglossus muscle, leading to the collapse of the upper airway during attempted breathing. It was estimated that 5%–10% of the general population suffers from the disorder [56]. Each episode of apnea or hypopnea lasts for a minimum of 10 seconds, results in a 3–4% drop in blood oxygenation, and concludes with a brief, unconscious arousal from sleep. The apnea-hypopnea index (AHI) is a scale that indicates the severity of the syndrome. It is the number of times the patient experiences apnea or hypopnea during one night, divided by the hours of sleep. OSA is categorized based on the AHI: an AHI of 5–15 indicates mild OSA, 15–30 signifies moderate OSA, and more than 30 represents severe OSA. The condition is worsened by factors such as alcohol consumption, sedative use, and weight gain. Obesity stands as one of the primary risk factors for OSA [57, 58].

Untreated OSA could lead to cardiovascular, metabolic, and cerebrovascular comorbidity. It has been associated with increased risks of hypertension, coronary artery disease, heart failure, and arrhythmias [59]. The pathophysiology of these complications is not entirely elucidated but seems to involve multiple pathways, one of which is endothelial damage due to oxidative stress.

Hypoxemia is considered the primary factor that leads to cardiovascular damage [60]. OSA is linked to nocturnal hypoxemia, leading to increased sympathetic outflow, systemic inflammation, and oxidative stress, all of which can damage the cardiovascular system [61].

The repeated strain on the cardiovascular system from these processes heightens the risk for serious cardiac events, including myocardial infarction and stroke. OSA is also known to worsen existing cardiovascular risk factors, including hypertension, obesity, and diabetes. These factors, combined with the metabolic disturbances associated with OSA, such as insulin resistance and dyslipidemia, further exacerbates cardiovascular risk. Importantly, the association between OSA and metabolic syndrome amplifies the likelihood of developing atherosclerosis and cardiovascular complications [62].

The evidence implicating oxidative stress as an important component of OSA pathophysiology has been consistently rising over the years. The chain of events promoting oxidative stress is most likely initiated by repeated breathing cessation, accompanied by drastic changes in oxygen tension. Intermittent hypoxia (IH) is thought to lead to oxidative stress by decreasing antioxidant mechanisms in periods of hypoxia and increasing reactive oxygen species (ROS) production during periods of re-oxygenation; termed an ischemia-reperfusion injury [63].

Oxidative stress may lead to hypertension via increased brain nuclei sympathetic activation and increased angiotensin II [64], and endothelial dysfunction which is thought to be a precursor of atheroma formation. Endothelial dysfunction under conditions of IH may be dependent on inflammation and oxidative stress as the anti-inflammatory drug infliximab, and the antioxidant drug L-glutathione, both blocked this impairment [65].

Endothelial dysfunction, a condition marked by an imbalance between vasoconstricting and vasodilating factors in the endothelium, may serve as a critical connection between stress and myocardial dysfunction in TTS [66]. This suggests that transient myocardial ischemia, followed by stunning, could be responsible for the characteristic reversible LV dysfunction. Additionally, endothelial dysfunction may account for the higher prevalence of TTS in postmenopausal women, as they exhibit both age-related and estrogen deficiency-related abnormalities in coronary vasomotor function [67]. Recent findings indicate that the majority of TTS cases occur in patients with a range of comorbidities, such as neurological, psychiatric, pulmonary, kidney, liver, and connective tissue disorders [68]. This association raises the possibility that these conditions may represent previously unrecognized predisposing factors for TTS.

**Fig. 2.**
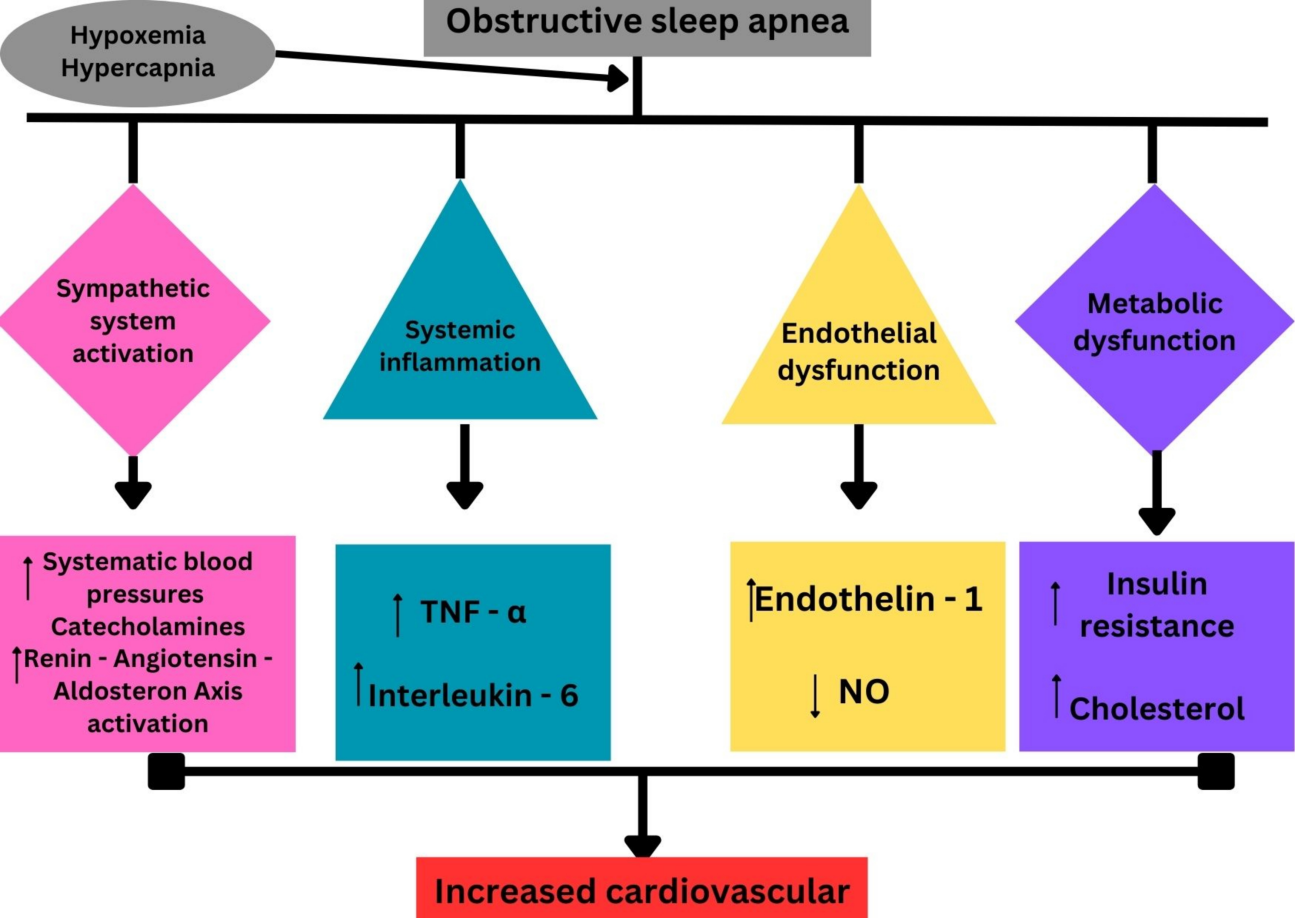
**The multifactorial pathogenesis of OSA and how their effect may increase the risk of cardiovascular disease**. OSA, obstructive sleep apnea; TNF-α, tumor necrosis factor alpha. Fig. 2 was created in the canva.com program.

The surge in catecholamines, triggered by recurrent hypoxia and sympathetic activation in patients with sleep-disordered breathing (SDB), results in myocardial damage through various mechanisms. These include direct catecholamine toxicity, adrenoceptor-mediated injury, epicardial and microvascular coronary vasoconstriction and/or spasm, and increased cardiac workload. This damage manifests functionally as transient apical left ventricular ballooning [69]. This link underscores the importance of addressing sleep-disordered breathing as both a contributor to hypertension and a potential trigger for acute cardiac events like TTS (Fig. 2).

### 5.3 Restless Leg Syndrome and TTS

Periodic limb movements during sleep (PLMS) involve repetitive, highly stereotyped movements of the limbs, most often the legs, that occur during sleep. Symptoms commonly associated with this condition include excessive daytime sleepiness (EDS), non-restorative sleep, nighttime awakenings, and/or insomnia. When these symptoms meet the diagnostic criteria outlined in the International Classification of Sleep Disorders, Third Edition (ICSD-3), the condition is classified as periodic limb movement disorder (PLMD) [70].

RLS and TTS are two distinct conditions that significantly impact patient health. RLS is a sensorimotor disorder characterized by an urge to move the legs, often accompanied by uncomfortable sensations. RLS affects approximately 5–15% of the general population, causing disrupted sleep and reduced quality of life. Finally, PLMS frequently occurs in patients suffering from OSA [71].

The relationship between RLS and cardiovascular disease (CVD) has been explored in both cross-sectional and prospective studies. A multivariate analysis from the Wisconsin Sleep Cohort revealed an association between RLS and CVD (odds ratio (OR) = 2.6, 95% CI 1.4–4.8) [72], but this was only observed in patients experiencing daily symptoms. Similarly, the Sleep Heart Health Study (n = 2546, mean age = 68) identified a link between RLS and CVD (OR = 2.4, 95% CI 1.6–3.7), which was also limited to individuals with symptoms occurring more than 16 days per month [73]. In contrast, the Women’s Health Study (n = 30,262 women, mean age = 64) did not find a significant relationship between RLS and overall CVD (OR = 0.98, 95% CI 0.74–1.3), but it did report a significant positive association with coronary revascularization (OR = 1.4, 95% CI 1.1–1.8) [74].

Extensive cross-sectional observational studies have revealed that both RLS and/or PLMS are linked to an approximately twofold higher risk of coronary artery disease and other cardiovascular conditions, such as heart failure, myocardial infarction, and hypertension [75, 76, 77]. Li *et al*. [78] found that this association persists even after adjusting for confounding cardiovascular risk factors, particularly in individuals with more severe or frequent RLS symptoms.

Given the established link between RLS and cardiovascular risk, it is important to explore the underlying mechanisms that may contribute to this association. One such mechanism involves the dysregulation of the HPA axis, which plays a critical role in stress responses and has been implicated in both RLS and TTS. The HPA axis regulates the release of stress hormones such as cortisol, which can influence both sleep quality and cardiovascular health. In RLS, chronic activation of the HPA axis may exacerbate symptoms by promoting a state of hyperarousal and disrupting sleep architecture. This dysregulation may also contribute to the heightened sympathetic activity observed in RLS patients, which could predispose them to cardiovascular events, including TTS.

In the context of TTS, the HPA axis plays a critical role in the release of stress hormones, such as catecholamines, which are known to trigger the myocardial stunning characteristic of TTS. Dysregulation of the HPA axis, as seen in RLS, could therefore exacerbate the release of these stress hormones, increasing the risk of TTS in susceptible individuals. This connection underscores the importance of understanding how sleep disturbances, such as RLS, may contribute to the development of TTS through shared mechanisms involving the HPA axis and sympathetic nervous system activation.

While the exact cause of RLS is still not fully understood, several pathophysiologic mechanisms have been implicated, including dopaminergic dysfunction, iron deficiency, genetic predisposition, and altered central nervous system (CNS) processes. Below, we explore these mechanisms:

Key pathophysiological mechanisms:

(i) Dopaminergic dysfunction: One of the most recognized mechanisms in RLS is impaired dopamine signaling in the brain, particularly within the basal ganglia [79]. Patients treated with low-dose dopaminergic medications showed an improvement in RLS symptoms [80, 81], while worsening of RLS symptoms was observed in patients administered dopamine antagonists [82]. The requirement for dopaminergic agonists to cross the blood-brain barrier (BBB) to alleviate RLS symptoms suggests that the dopaminergic system within the central nervous system, rather than the peripheral nervous system, plays a key role in the pathophysiology of RLS [83].

(ii) Iron deficiency: Brain iron deficiency is a well-established factor that impacts dopamine synthesis and function. Iron is an essential cofactor for the synthesis of dopamine, and iron deficiency has been closely linked to RLS [84]. Reduced iron levels in the brain, particularly in the substantia nigra and other dopaminergic areas, contribute to impaired dopamine production and function [85]. In a population of patients with iron-deficient anemia, the prevalence of RLS was reported to reach up to 31.5% [86]. This prevalence is approximately six times higher than that observed for RLS in the general population [87]. However, the majority of RLS patients do not present with systemic iron deficiency.

(iii) Genetic factors: Certain genes, such as myeloid ecotropic viral integration site 1 (*MEIS1*) and broad-complex-tram-track-bric-a-brac domain 9 (*BTBD9*), have been linked to RLS susceptibility. Familial forms of RLS suggest autosomal dominant inheritance patterns, and genome-wide association studies (GWAS) have identified genes such as *MEIS1*, *BTBD9*, and *MAP2K5*/*SKOR1* [88].

*MEIS1* gene: Variants in the *MEIS1* gene have been strongly associated with RLS and are thought to influence neural development and dopamine regulation.

**Fig. 3.**
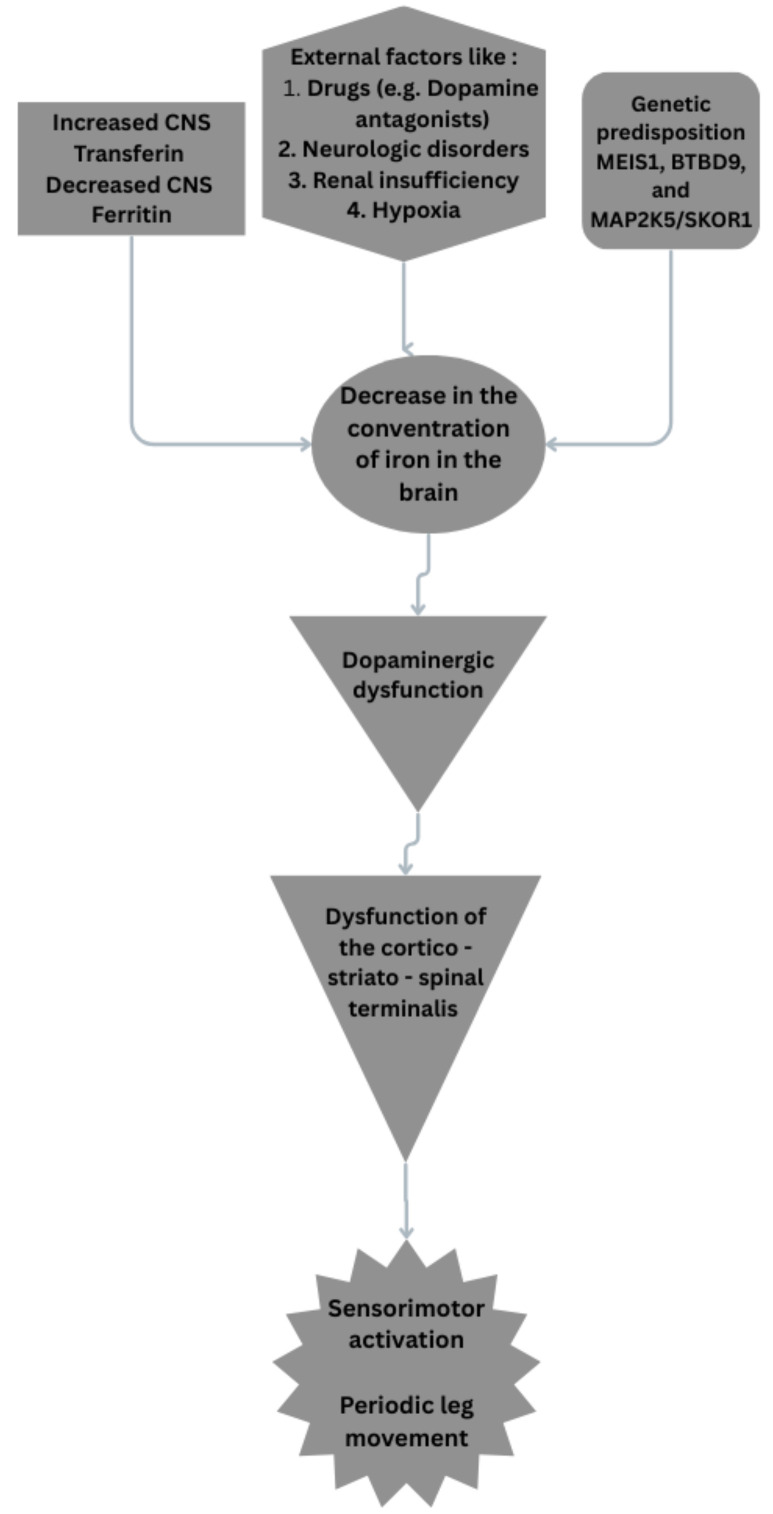
**Possible pathophysiologic mechanisms on RLS**. RLS, restless leg syndrome; MEIS1, myeloid ecotropic viral integration site 1; BTBD9, broad-complex-tram-track-bric-a-brac domain 9. Fig. 3 was created in the canva.com program.

*BTBD9* gene: This gene is involved in iron metabolism, and its variations have been linked to altered iron homeostasis, further supporting the iron-dopamine hypothesis.

(iv) Autonomic nervous system (ANS) involvement: Evidence suggests that ANS dysfunction could contribute to the sensory and motor symptoms experienced in RLS. This includes alterations in heart rate variability and stress responses [89] (Fig. 3).

(v) Role of the HPA axis and its dysregulation can exacerbate RLS symptoms by promoting a state of chronic stress and arousal. Elevated cortisol levels can interfere with sleep and potentially worsen the underlying dopaminergic dysfunction, creating a vicious cycle of poor sleep and increased symptom severity. Specifically, the activation of the HPA axis leads to the release of corticotropin-releasing hormone (CRH) from the hypothalamus, which triggers the anterior pituitary to release adrenocorticotropic hormone (ACTH). ACTH then stimulates the adrenal cortex to produce cortisol.

**Fig. 4.**
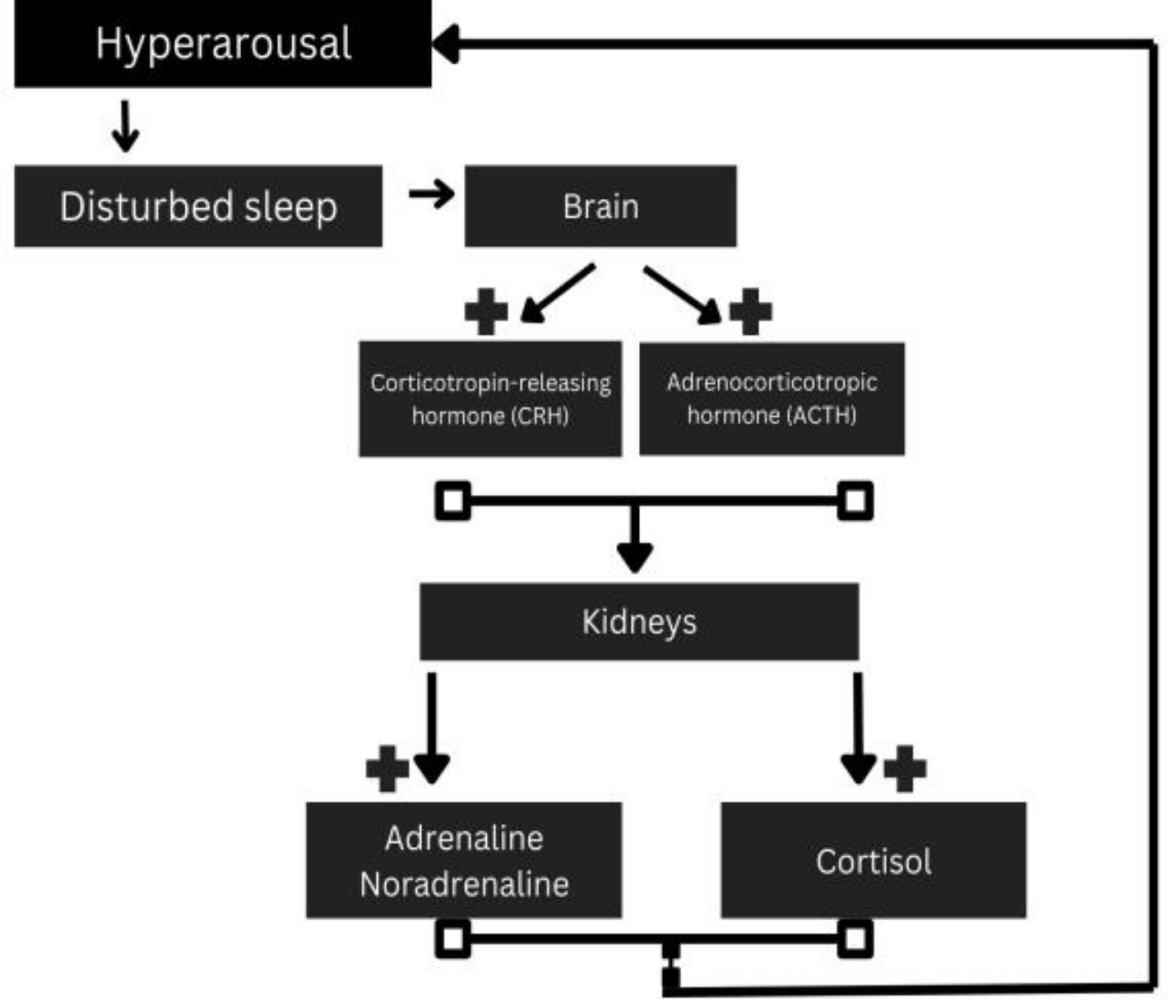
**HPA axis and its correlation with hyperarousal**. HPA, hypothalamic-pituitary-adrenal. Fig. 4 was created in the canva.com program.

This cascade is a key component of the body’s stress response [90] (Fig. 4).

Research suggests that individuals with RLS may have an altered stress response, marked by chronic low-level HPA activation. This condition can exacerbate nighttime restlessness and discomfort in the legs, contributing to the cycle of disturbed sleep and increased stress. Elevated cortisol levels, particularly at night, can further disrupt sleep architecture and amplify symptoms of RLS.

The sensory and motor symptoms of RLS are thought to result from a hyperexcitable state in the CNS. This hyperexcitability may involve altered thalamocortical pathways and spinal cord activity.

Hyperexcitability in the spinal cord: Studies suggest that increased excitatory neurotransmission in the spinal cord, potentially involving glutamate, contributes to the motor restlessness experienced in RLS [91, 92, 93]. Allen *et al*. [94] reported in their study that magnetic resonance spectroscopy imaging in RLS patients revealed a significant rise in thalamic glutamate levels, which was associated with the amount of time spent awake during the sleep period. These findings suggest a presynaptic hyperglutamatergic state in RLS, which may contribute to the hyperarousal characteristic of the condition.

Cortical and subcortical networks: Functional magnetic resonance imaging (MRI) studies have shown abnormal activation in regions such as the primary motor cortex and somatosensory pathways in RLS patients, suggesting that CNS dysregulation contributes to both the sensory and motor aspects of the disorder [89].

Over the past 15 years, numerous studies have indicated a connection between RLS and heart disease, stroke, and hypertension. RLS patients often exhibit a nondipping pattern in nocturnal blood pressure, a well-established risk factor for CVD [95]. In an analysis involving 57,417 female participants from the Nurses’ Health Study (NHS), RLS was significantly associated with a 43% higher risk of future CVD-related mortality [96]. In a recent cohort study with a follow-up period exceeding three years, the presence of RLS was linked to an increased risk of developing CVD. Compared to the non-RLS group, the adjusted hazard ratio (HR) was 1.26 (95% CI 1.20–1.32; *p* < 0.001) for the RLS group receiving treatment and 1.53 (95% CI 1.42–1.65; *p* < 0.001) for the RLS group without any treatment, after adjusting for potential confounders. Among RLS patients, treatment was associated with a 13% lower risk of CVD (95% CI 4%–20%; *p* < 0.001) compared to those without treatment. A significantly reduced CVD risk was observed across all RLS treatment types, including dopaminergics, anticonvulsants, benzodiazepines, and opiates (adjusted HR range, 0.71–0.84; *p* < 0.001 for all) [97].

While direct studies linking RLS and TTS are lacking, there is compelling evidence that shared mechanisms involving ANS dysregulation and HPA axis activation could link these disorders. In conclusion TTS and RLS share common ANS dysregulation, HPA axis activation and sleep disturbances. Further investigation into how these systems interact and predispose individuals to both conditions could improve patient care and guide preventive measures.

### 5.4 Sleep Deprivation and TTS

According to recent outcome-based recommendations from the National Sleep Foundation, adults should aim for a sleep duration of 7 to 9 hours per night [98]. Recent data indicate that only 48% of the U.S. adult population reports sleeping within the recommended range of 7 to 9 hours per night [99], while 26% average 6 to 7 hours of sleep, and 20% sleep less than 6 hours nightly. This shift in sleep patterns appears to be linked to demanding work environments and lifestyle changes, including increased exposure to artificial lighting and the widespread use of new communication technologies.

In addition to insomnia and OSA, sleep deprivation is another significant sleep disturbance that may contribute to the development of TTS. Many patients experiencing TTS report preceding periods of sleep deprivation, which can lead to heightened sympathetic nervous system activity, oxidative stress, and cardiovascular strain—key factors in the pathophysiology of TTS. The mechanisms linking sleep deprivation to TTS include:

(1) Heightened reactivity to stress: Sleep deprivation (SD) exerts varying effects on cardiovascular stress responses between individuals. Emotional stability (ES) is a personality trait pertinent to SD and stress responding. This amplified stress can trigger the TTS cascade in vulnerable individuals [100].

(2) Reduced resilience to physical stressors: Sleep is essential for the proper regulation of cardiac functions in both healthy individuals and those with medical conditions [101]. The quality and duration of sleep can significantly impact cellular immunity, and even brief periods of sleep deprivation may compromise the immune system [102].

Insufficient sleep leads to higher levels of cortisol, often referred to as the stress hormone. Elevated cortisol levels can promote insulin resistance, inflammation, and hypertension, all of which are recognized risk factors for CVD. In the context of TTS, an acute, excessive catecholamine surge is central to the myocardial stunning observed. SD, therefore, acts as both a direct and indirect contributor [103, 104]. It is plausible that during an emotional event, a sudden surge in catecholamines may have a distinct impact compared to the chronic release of catecholamines associated with a physical trigger [105].

Evidence suggests that both prolonged sleep restriction and extremely limited sleep opportunities are linked to increased levels of plasma or urinary norepinephrine (NOR). In the study by Covassin *et al*. [106], the rise in plasma NOR was accompanied by elevated 24-hour systolic blood pressure, but this effect was observed only in women [107].

While most research on sleep deprivation or restriction has focused on plasma NOR, Grimaldi and colleagues [108] examined 24-hour urinary NOR following eight consecutive days of 5-hour sleep restriction. Notably, this study included a second phase that replicated the 8-day, 5-hour sleep restriction but also introduced circadian misalignment by delaying the sleep opportunity by approximately 8 hours on half of the nights. While sleep restriction alone did not significantly affect urinary NOR, the combination of sleep restriction and circadian misalignment led to a marked increase in 24-hour urinary NOR. This increase was particularly pronounced during sleep and early morning hours, periods associated with a higher risk of adverse cardiovascular events. Similar to other studies discussed in this review, this research is limited by a predominantly male study population, preventing an exploration of potential sex-based differences.

Further studies are needed to investigate the relationship between sleep deprivation/restriction, sympathetic activity, and sex differences, as the potential mediating role of sex in the connection between sleep restriction with circadian misalignment and increased urinary NOR remains unclear. Additionally, it is yet to be determined whether behavioral sleep extension interventions reduce sympathetic activity through direct or indirect mechanisms and whether these effects differ between men and women [109].

## 6. Clinical Implications

Understanding the connection between sleep disorders and TTS has significant clinical implications. Given the increased risk of cardiovascular morbidity associated with conditions like OSA and insomnia, early recognition and treatment of these sleep disorders could potentially reduce the incidence of TTS. Polysomnography, the gold standard for diagnosing OSA, should be considered in patients with unexplained cardiomyopathy, particularly those with risk factors for sleep apnea.

Understanding the connection between insomnia and TTS highlights the need for a holistic approach to treating insomnia, particularly among individuals at risk for cardiovascular issues. Cognitive behavioral therapy for insomnia (CBT-I) has been shown to improve sleep quality and reduce stress response, potentially mitigating the cardiovascular risks associated with insomnia. Pharmacological treatments, including beta-blockers or anxiolytics, could also contribute to mitigating the cardiovascular impacts of insomnia, although further research is necessary to assess their long-term effectiveness.

Therapeutic interventions should be initiated immediately upon diagnosing OSA. Continuous positive airway pressure (CPAP) is considered the gold standard for treating moderate to severe OSA [110]. In patients suffering from mild and moderate OSA or those who cannot tolerate the CPAP, oral applications are recommended [111]. Surgical intervention should be considered as a supplementary treatment to CPAP once the site of obstruction has been identified. Procedures such as septoplasty, turbinate reduction, nasal valve surgery, and sinus surgery are performed to address nasal obstruction associated with OSA [112]. Cognitive-behavioral therapy for insomnia, could mitigate sympathetic activation and lower the risk of cardiovascular complications, including TTS [113].

When RLS is suspected based on clinical presentation, the diagnostic process should include evaluating serum ferritin, transferrin saturation, and, in certain cases, a soluble transferrin receptor assay to assess for iron deficiency or low body iron stores, which may worsen RLS symptoms. Iron-replacement therapy is recommended when transferrin saturation falls below 20–25%, even if ferritin levels are normal or elevated. Non-pharmacologic approaches, such as massage, stretching, walking, cognitive distraction (e.g., playing games or solving puzzles), or temperate baths, may provide temporary relief, but their benefits are often short-lived and lack strong evidence despite anecdotal support. For individuals with intermittent or mild symptoms, the initial therapeutic approach typically involves selecting an appropriate monotherapy. The iron-deficiency hypothesis remains central to understanding RLS pathophysiology, making oral iron-replacement therapy the first-line treatment. Adding vitamin C can enhance the absorption of elemental iron from the gastrointestinal tract and often reduces adverse effects. Recently, gabapentin and related medications (e.g., gabapentin enacarbil, pregabalin [Lyrica]) have emerged as preferred first-line options for managing RLS [114]. Moreover, dopamine agonists (pramipexole, ropinirole, rotigotine) have been the traditional mainstays of therapy for RLS.

Given the potential role of sleep disorders in precipitating TTS, preventive measures should be emphasized:

(i) Promotion of healthy sleep habits: Educating patients about the importance of sleep hygiene and stress management.

(ii) Managing comorbid conditions: Treating related conditions like hypertension, obesity, and diabetes, which can contribute to both sleep disorders and cardiac risks.

(iii) Lifestyle modifications: Promoting habits like regular physical exercise and stress management techniques can enhance both sleep quality and cardiovascular health.

(iv) Given the connection between TTS and sleep disorder is of paramount importance to recognize the symptoms and treat as fast as possible so that the detrimental effects of the former will be prevented. Additionally, it is important to use the Intermark score to stratify our TTS patient, so that both our diagnostic approach and treatment take place without delay.

## 7. Conclusions

Sleep disorders, particularly OSA, insomnia, and RLS, appear to play a contributory role in the development of TTS. The shared mechanisms of heightened sympathetic nervous system activity, oxidative stress, and cardiovascular strain in both sleep disturbances and TTS suggest a common pathway that may precipitate the syndrome. Furthermore, dysregulation of the HPA axis, as seen in conditions like RLS and sleep deprivation, may exacerbate the release of stress hormones, further increasing the risk of TTS in individuals with sleep disturbances. While more research is needed to fully elucidate the exact relationship between sleep disorders and TTS, early identification and management of sleep disorders may offer a novel approach to preventing this acute form of cardiomyopathy. Recognizing and addressing sleep disorders in TTS patients can aid in comprehensive care, potentially reducing recurrence and improving overall cardiovascular health. Clinicians should integrate sleep assessments into cardiovascular evaluations and consider targeted interventions to optimize outcomes.
